# Abuse of Quinine

**Published:** 1890-08

**Authors:** Wm. Steinrauf

**Affiliations:** St. Charles, Mo.


### ABUSE OF QUININE.

WM. STEINRAUF, M. D., ST. CHARLES, MO.

Seeing that one of the contributors of THE HOMŒOPATHIC PHYSICIAN asks for an article upon the evils of Quinine in massive doses, I submit the following few words on the subject.

I have always practiced medicine in what is generally called a "malarial" country and where allopathy reigns supreme, where Homœopathy is decried as quackery, and a homœopathic physician is looked upon as a crank. Such is the State of Missouri. Quinine, Calomel, and Morphine are the trinity of the old-school doctors' armamentarium in this part of the country. I believe that Quinine is the worst of the lot, because used three times as often as any of the others. Fully one-half of the chronic diseases that homœopathic physicians in this part of the State are called upon to treat, have become chronic because of the continued and excessive use of Quinine, and, I think, it is so all over the land. From an aching of the big toe to the last stage of consumption, Quinine is the Alpha and Omega of the scientific treatment of our old-school doctors. The congested livers, bloated abdomen, and hypertrophied spleens tell a plain story.

Our allopathic doctors, who say that the experience of ages and all scientific knowledge are on their side, claim that Quinine, in large doses, is the true and only remedy that can be employed with success in "malaria." And as more than one-half of all diseases have a malarious bottom, Quinine in large and frequently-repeated doses is the sheet-anchor. That is the way they argue. But "malaria," thus suppressed, is not cured. Hear what Hahnemann says: "True, they can no longer complain that the paroxysm of their original disease reappears on certain days and at certain hours; but note the earthy complexion of their puffy faces, the dullness of their eyes!

"See how oppressed is their breathing, how hard and distended is their epigastrium, how tensely swollen their loins, how miserable their appetite, how perverted their taste, how oppressed and painful their stomachs by all food, how undigested and abnormal their fæcal evacuations, how anxious, dreamful, and unrefreshing their sleep. Look how weary, how joyless, how dejected, how irritable, sensitive, or stupid they are as they drag themselves about, tormented by a much greater number of ailments than afflicted them in their ague. And how often does not such a China-cachexy often last in comparison with which death itself were often preferable?

"Is this health? It is not ague, that I readily admit, but confess, and no one can gainsay it, it is certainly not health. It is rather another, but a worse disease than ague. It is the China-disease, which must be more severe than the ague, otherwise it could not overcome and suppress (suspend) the latter.

"Should the organism, as it sometimes will, recover from the China-disease after many weeks, then the ague, which has till now remained suspended by the superior force of the dissimilar China-disease, returns in an aggravated form, because the organism has been so much deteriorated by the improper treatment."

Let us mention a case or two plainly showing the terrible effects of this drug on the system.

Mrs. K., wife of a miller, has been complaining for the last eight or ten years. She has aches and pains in different parts of the body; feels tired and worn; no ambition to do anything; no appetite, and always constipated. The diagnosis of our friend the enemy was "malaria." Now constipation always indicates cathartics with them, and malaria invariably Quinine, just as pain points to Opium, syphilis to Iodide of Potassium in large and increasing doses.

This good woman has now taken these two—cathartics and Quinine, especially the latter—off and on for many years, and still she is not well, still the cry is, "malaria." Her liver began to swell, and a terrible pain in her right side and below the right shoulder-blade supervened. Insomnia was now a prominent feature, and the woman began to fear that she might eventually loose her reason.

In this dilemma I was asked to take charge of the case after her allopathic adviser had freely acknowledged his failure to cure. As I could see no particular indication for any particular remedy, Sac-lac. was given for a few days, when she had the following symptoms: Dizziness, roaring in the ears, tired and given-out feeling. Besides, there was constipation and belching of wind and no sleep. Nux-vom.$^{cm}$, one dose. This relieved the constipation, but all the other symptoms remained. Nux-vom.$^{cm}$ again. Within two weeks very little change. Carbo-veg.$^{cm}$.

After another two weeks there was indeed a wonderful change for the better. The lady, that a few weeks before looked haggard and icteroid, was sleepless and would not venture out-of-doors, now became quite lively, ate, looked bright, slept well and began to stroll about her premises. She was under treatment for about three months, Nux-vom., Carbo-veg., and Sulphur made a new woman of her. She freely and loudly proclaims that it was the continuous use and abuse of Quinine that was the cause of all her aches and pains these years. She was never without taking a few doses of the drug every week, and whenever her condition would be in the least aggravated, her physician would claim that the dose must be increased. She is a small and frail woman, of a mild and sympathetic temperament, and how her system withstood this battle with the Quinine from year to year is indeed a marvel.

Chas. B. has had the third-day ague six years ago, and after doctoring more than a year, finally succeeded in getting it suppressed with a prescription that called for forty grains of Quinine, and one-half grain of Arsenic three times a day for four days. For too or three months after losing his ague, he began to get peculiar hallucinations, especially in the spring-time and in the fall. The spells would come on toward evening. Could work during the day. When the spells came on he complains of terrible pains in the left hypochondrium, runs about and acts strangely. The family and all the neighbors think him insane when one of these attacks occur. He will then not sleep all night, but still be able to work during the day. He never threatens to do violence to any one, but has frequently talked of committing suicide when this pain is at its height. He neither uses tobacco, nor does he drink. He dates his troubles from the time he used said large and excessive doses of Quinine and Arsenic. Nux-vom.$^{cm}$, one dose every three hours till three doses are taken. Sac-lac. In one week the spells had grown very much less. Sleeps better. Sac-lac. another week. Improvement more marked. Bowels, heretofore very costive, now quite regular. Sac-lac. for the next four weeks. Now an eruption appeared on the whole body, for which he took Sulphur DMM, Swan. Six weeks later he was well and has remained so now over ten months.

So much for the terrible abuse of Quinine. I have treated many such cases, but have not every time come off with flying colors. In my estimation the majority are never cured.

